# T-Bet Expression Mediated by the mTOR Pathway Influences CD4^+^ T Cell Count in Mice With Lethal *Candida* Sepsis

**DOI:** 10.3389/fmicb.2020.00835

**Published:** 2020-05-05

**Authors:** Guangxu Bai, Hao Wang, Wen Han, Na Cui

**Affiliations:** ^1^Department of Critical Care Medicine, Peking Union Medical College Hospital, Peking Union Medical College, Chinese Academy of Medical Sciences, Beijing, China; ^2^Department of Clinical Laboratory, Peking Union Medical College Hospital, Peking Union Medical College, Chinese Academy of Medical Sciences, Beijing, China; ^3^Beijing Key Laboratory for Mechanisms Research and Precision Diagnosis of Invasive Fungal Diseases, Beijing, China

**Keywords:** T-bet, CD4^+^ T cell count, lethal *Candida* sepsis, Murine Sepsis Score, mammalian target of rapamycin

## Abstract

The sustained high morbidity and mortality of *Candida* sepsis are mainly caused by compromise of host immunity. Clinically, it is often manifested as a significant decrease in CD4^+^ T cell count, although the mechanism is unclear. We established a lethal mice *Candida* sepsis model and used Murine Sepsis Score to group mice with different disease severity to establish the influence of T-bet expression on CD4^+^ T cell count in *Candida* sepsis. We found that CD4^+^ T cell count decreased in *Candida*-infected compared to uninfected mice, and the degree of decrease increased with aggravation of sepsis. Expression of T-bet similarly decreased with worsening of sepsis, but it was significantly enhanced in candidiasis in comparison of naïve state. To clarify its possible mechanism, we measured the activity of mammalian target of rapamycin (mTOR), which is a key regulator of T-bet expression. The mTOR pathway was activated after infection and its activity increased with progression of sepsis. We used mice with T-cell-specific knockout of *mTOR* or tuberous sclerosis complex (*TSC*)1 to further inhibit or strengthen the mTOR signaling pathway. We found that *mTOR* deletion mice had a higher CD4^+^ T cell count by regulating T-bet expression, and the result in *TSC1* deletion mice was reversed. These results demonstrate that T-bet expression mediated by the mTOR pathway influences the CD4^+^ T cell count in mice with *Candida* sepsis.

## Introduction

*Candida albicans* is one of the common commensals in humans. However, for immunocompromised and critically ill patients, infection with *C. albicans* can have disastrous consequences (Kullberg and Arendrup, 2015). Candidemia is the fourth most common bloodstream infection in intensive care units, and mortality of *Candida* bloodstream infection is close to 40%, which is significantly higher than mortality of sepsis caused by most bacterial pathogens (Wisplinghoff et al., 2004). Septic shock induced by *C. albicans* can be lethal, with an estimated mortality rate of nearly 90%; three times that of septic shock caused by bacteria (Guzman et al., 2011). Despite the use of antibiotics to which fungi are highly sensitive, and large-scale preventive trials, this situation has not been improved. The incidence and mortality of *Candida* sepsis remain high (Wenzel and Gennings, 2005; Pappas et al., 2016). Consequently, a lot of research has attempted to address this problem by immunoregulation or enhancing host immunity (Delsing et al., 2014; Sam et al., 2018).

It is well known that the intensity of the adaptive immune response is closely related to the occurrence, development and prognosis of *Candida* sepsis (Richardson and Moyes, 2015). The adaptive immune response mediated by CD4^+^ T cells is an essential pathway for the host to quickly eliminate invasive pathogens. A decreased CD4^+^ T cell count means that the host’s immune system is compromised and the intensity of the immune response is weakened, which are major reasons for poor outcomes in patients with *Candida* sepsis (Spec et al., 2016). Thus, understanding the molecular mechanisms that cause the decline of CD4^+^ T cell count in patients with *Candida* sepsis may provide new insights into improving outcomes.

T-bet is the most well-known specific transcription factor of the T-box family and regulates the differentiation and development of CD4 type 1 helper T (Th1) cells. T-bet directly promotes Th1 differentiation and inhibits differentiation of other Th cells; therefore, it has significant Th1 specificity, which ensures polarization of naïve T cells in the direction of Th1 (Szabo et al., 2000). Many studies have measured the expression of T-bet to reflect the level of CD4^+^ T cell differentiation and evaluate the host adaptive immune response after infection (Wu et al., 2013; Xu et al., 2019). In addition, T-bet expression is mainly regulated by the mammalian target of rapamycin (mTOR) signaling pathway. As an evolutionarily conserved signaling pathway, mTOR is widely involved in lymphocyte biology. When *Candida* infection occurs, mTOR is activated, integrating numerous environmental and immune elements, and participates in regulation of lymphocyte development, activation, differentiation and death (Ben-Sahra and Manning, 2017; Mossmann et al., 2018). We have demonstrated that mTOR is involved in CD8^+^ T cell differentiation that is achieved by regulating expression of transcription factors (Cui et al., 2016; Wang et al., 2018).

Hence, we hypothesized that the mTOR pathway might mediate expression of specific transcription factor T-bet to regulate lymphocyte differentiation during *Candida* sepsis and influence CD4^+^ T cell count, leading to compromise of host immune function.

## Materials and Methods

### Pathogen Preparation

*Candida albicans* SC5314 was kindly provided by the Department of Clinical Laboratory, Peking Union Medical College Hospital. *C. albicans* was grown overnight in YPD broth at 37°C. We harvested the cells by centrifugation (1 min, 800 × *g*, room temperature), washed them with sterile pyrogen-free phosphate-buffered saline (PBS) twice, and resuspended the cells in culture medium (Heather et al., 2014). Finally, we used the turbidity method (Espinel-Ingroff and Kerkering, 1992) to adjust the concentration of conidia to 106 colony-forming units (cfu).

### Mice

Male healthy C57BL/6 N mice aged 4–5 weeks weighing 16–18 g were bred under specific pathogen-free conditions in the Animal Laboratory of Peking Union Medical College Hospital. *TSC1*^loxp/loxp^, *mTOR*^loxp/loxp^ and *Lck-cre* mice were generously given by Dr. Yong Zhao (State Key Laboratory of Biomembrane and Membrane Biotechnology, Institute of Zoology, Chinese Academy of Sciences, Beijing, China). Our experiments were approved by the Ethics Committee of Peking Union Medical College Hospital, Chinese academy of medical sciences (Beijing, China) and the approval No is JS-1170. We cross-bred *TSC1*^loxp/loxp^ and *mTOR*^loxp/loxp^ mice with mice expressing Cre recombinase, respectively, and thus obtained F1 generation *Lck-cre; TSC1*^loxp/–^ and *Lck-cre; mTOR*^loxp/–^ mice. After that F1 generation mice were interbred in the group and F2 generation *Lck-cre; TSC1*^loxp/loxp^ and *Lck-cre; mTOR*^loxp/loxp^ mice were obtained. There were 30 mice in each group. Before the experiment, all mice were adapted to a 12-h day–night cycle for >1 week and the food and water were given *ad libitum*.

### *C. albicans* Infection Model

Wild-type (WT) mice and gene-specific knock-out mice were all injected intravenously through the tail vein with the same dose of *C. albicans* conidia (1 × 10^6^ cfu/mouse) in 100 μl PBS (Heather et al., 2014) Uninfected control mice were injected with the same dose of saline. Since renal fungal burden at 12 h after challenge has been shown associated with the severity of the disease model (Hurtrel et al., 1980), all mice were euthanized at 12 h after infection and the kidneys, lungs, spleens and livers were removed for histological analysis with Gomori’s methenamine silver (GMS) staining.

### Murine Sepsis Score

The *Candida* intravenous challenge is one of the common models to mimic clinical candidemia. However, because of the different characteristics of the mice and strains used, the severity of many models is not consistent (MacCallum and Odds, 2005; Carreras et al., 2019). In order to ensure the consistency of the model, we used the Murine Sepsis Score (MSS) for severity evaluation (Shrum et al., 2014). MSS includes spontaneous activity, response to touch and auditory stimuli, posture, respiration rate and quality, and appearance in five sections. Each section is assessed on a scale of 0–4 points (Parameters of MSS was provided in Supplementary Material). Mice with >3 points were demonstrated to have sepsis and those with >10 points were associated with a significant increase in mortality. Therefore, at 12 h after challenge, we collected relevant data and performed the MSS on each mouse.

### Histopathology

Kidneys, spleens, livers and lungs were stored in 10% neutral-buffered formalin at 4°C after removing from anesthetized and sacrificed mice. After embedding in paraffin and cutting into 4-μm slices, the tissues were stained with GMS. We used Pannoramic DESK (3D HISTECH; Hungary) to scan and image the pathological sections stained with GMS.

### Lymphocyte Isolation and Cell Counting

To determine the T-cell count, we took the spleens of the mice, torn up them with eye tweezers and washed with PBS, then used ammonium chloride–potassium lysis buffer (BD) to lyse the red blood cells in the spleens. After that, we resuspended the centrifuged spleen cells (4°C, 500 *g*, 5 min) in RPMI-1640 medium, used trypan blue to stain the cell suspension and quantified their viability and number with a TC20 automated cell counter (Bio Rad).

### Surface Marker Staining and Cell Sorting

Based on the PBMCs of spleen, we detected the ratio of CD4^+^ T cells. The cell suspension density was adjusted to 1 × 10^6^/ml for flow cytometry and initially gated by forward and side scatter properties (Gating strategy was provided in Supplementary Material). CD4^+^ T-cells and CD8^+^ T-cells were then gated. Then we sorted CD4^+^ T cells and the purity of CD4^+^ T cells was also detected. The sorted CD4^+^ T cells were then used for western blotting. The machine we used is BD FACS AriaIII. In the process of cell sorting we used doublet exclusion to ensure the flow cytometry detection of individual cells. The CD4 Monoclonal Antibody (RM4-5), PerCP-Cyanine 5.5, CD8 alpha Monoclonal Antibody (53-6.7), FITC (MA1-10303) added in cell suspension were purchased from eBioscience. The purity was verified as >90%. The sorted cells were used for western blotting.

### Western Blotting

To assess the activation of mTOR during *Candida* sepsis, we used western blotting to detect the levels of p-mTOR and p70S6 kinase. As one of the downstream effector molecules of mTORC1, p70S6 kinase is widely involved in the regulation of mTOR signaling pathway on cellular functions, such as proliferation and differentiation, autophagy and apoptosis, and the transformation of glucose metabolism (Ghosh and Kapur, 2017). In addition, we detected the expression of T-bet through western blotting. First, we used 300 μl RIPA buffer to extract the protein from sorted CD4^+^ T cells on ice. Secondly, the sample was centrifuged at 12000 rpm at 4°C for 15 min. The supernatant was taken to measure the protein concentration by the bicinchoninic acid method. Thirdly, 30 mg protein was subjected to SDS-PAGE and transferred to polyvinylidene fluoride membranes and 5% nonfat milk was used for blocking the membranes and further incubated them overnight with specific primary antibodies. At last we washed the membranes with TBST buffer for three times, incubated with the corresponding horseradish-peroxidase-conjugated secondary antibody for 1 h and developed on X-ray film with chemiluminescence reagent. Images were captured by Bio-Rad ChemiDoc XRS+, and densitometric analysis was performed using Quantity-One software (Bio-Rad). We used the following antibodies: anti-p-p70S6 (Cell Signaling Technology), anti-phospho (p)-mTOR (Cell Signaling Technology) and anti-T-bets (Cell Signaling Technology); anti-glyceraldehyde-3-phosphate dehydrogenase (GAPDH) (Santa Cruz Biotechnology) was used as a control.

### Statistical Analysis

Data were analyzed using SPSS version 24.0 software (SPSS Inc., Armonk, NY, United States) (Relevant data was conducted a normality test and the results were provided in Supplementary Material). Two tailed Student’s *t*-test or one-way analysis of variance (ANOVA) followed by Dunn’s/Bonferroni’s test were used to determine the statistical significance of differences. *P* < 0.05 was considered statistically significant.

## Results

### Model Confirmation of Lethal Sepsis Induced by Intravenous Injection of *C. albicans*

**FIGURE 1:**
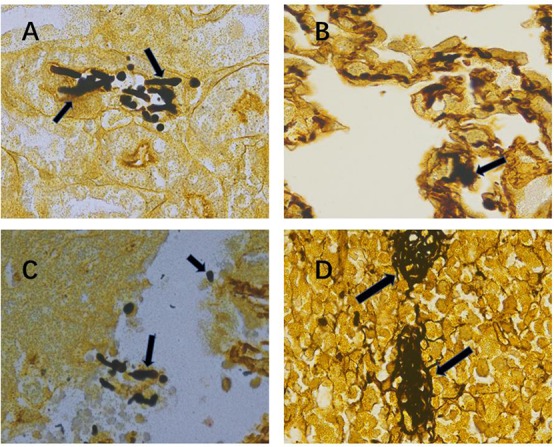
Confirmation of severe invasive *Candida albicans* multiorgan infection mouse model. Representative kidney **(A)**, lung **(B)**, liver **(C)** and spleen **(D)** histological sections at 12 h after intravenous injection of *C. albicans* were stained with Gomori’s methenamine silver to visualize *C. albicans* hyphae and spores. Black arrows indicate hyphae and spores. Magnification, 1000×.

In this study, we used the established mouse model of lethal *Candida* sepsis (Wang et al., 2019), which had been verified to express the characteristics of clinical *Candida* sepsis well. As shown in Figure 1, the hyphae and spores of *C. albicans* were significantly distributed in the tissues of infected mice.

### Use of MSS to Distinguish Different Degrees of Sepsis

**FIGURE 2:**
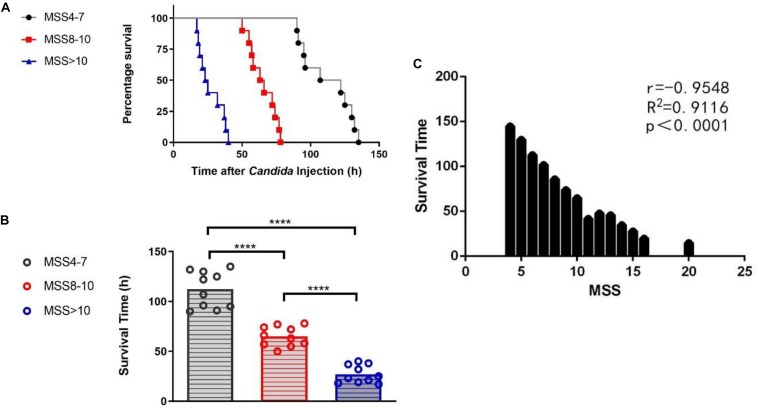
Group difference and correlation between Murine Sepsis Score (MSS) and survival time of mice. Survival rates between MSS 4-7, MSS 8-10 and MSS >10 mice **(A)**. Mice in different MSS group had varied survival time **(B)**. Correlation between MSS and survival time **(C)**. Survival rates were observed for every 1 h after *Candida* injection. *P*-value between any two of the three different curves of MSS groups analyzed by log-rank test was <0.0001. MSS was assessed at 12 h after *Candida* injection. Mean ± SD, 10 mice per group. ****P < 0.0001.

Although the mice were all inoculated with the same dose of *C. albicans* suspension, the survival time of the mice varied significantly, which implied different degrees of sepsis severity. Since MSS can judge the severity of sepsis to some extent, all mice were scored with MSS at 12 h after *C. albicans* injection. The differences of the survival curves between any two of three MSS groups were statistically significant (*P*-value between any two of the three different curves of MSS groups analyzed by log-rank test was <0.0001) (Figure 2A). Mice with different MSS had different survival time. Mice with MSS 4–7 had the longest survival time; mice with MSS >10 had the shortest survival time; and the survival time of the MSS 8–10 group was in the middle (Figure 2B). The MSS had significant negative correlation with the survival time (Figure 2C). These results demonstrate that MSS can distinguish different degrees of fungal sepsis.

### CD4^+^ T Cell Count and T-Bet Expression in *C. albicans*-Infected Mice With Aggravation of Sepsis

**FIGURE 3:**
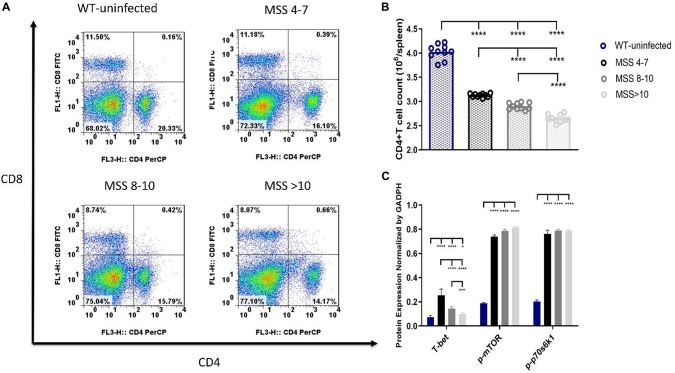
CD4^+^ T cell count, T-bet expression and mammalian target of rapamycin (mTOR) pathway activation differed according to degrees of sepsis. Splenocytes were obtained at 12 h after *Candida* injection. **(A,B)** CD4^+^ T cells were sorted by flow cytometry analysis. Protein levels of T-bet, phospho (p)-mTOR and p-P70S6 **(C)** in CD4^+^ T cells. Levels of T-bet, p-mTOR and p-P70S6 in CD4^+^ T cells were quantified by western blotting. The amount of each protein level was normalized by GAPDH. Mean ± SD, 10 mice per group, analyzed by one-way analysis of variance multiple comparisons. *P < 0.05, ***P < 0.001, ****P < 0.0001. WT, wild type; (Summarization of the flow cytometry scatter plots was provided in Supplementary Material).

To determine the effect of *Candida* sepsis on CD4^+^ T cells and its underlying mechanism, we separated CD4^+^ T cells from the spleen by flow cytometry and further measured the expression of T-bet by western blotting (Figure 3A). Compared with uninfected control mice, CD4^+^ T cell count significantly decreased in all three MSS groups which represented *C. albicans*-infected mice and continued to decrease with aggravation of sepsis (Figure 3B). The expression of T-bet also decreased with aggravation of sepsis but was significantly enhanced in candidiasis in comparison of naïve state (Figure 3C). These results demonstrate that T-bet might play an important role in regulation of CD4^+^ T cells during lethal fungal sepsis.

### mTOR Pathway Activation Was Elevated in *C. albicans*-Infected Mice

To assess the activation of mTOR, a key signaling pathway that regulates T-bet expression, in *Candida* sepsis, we used western blotting to detect the levels of p-mTOR and p70S6 kinase. Compared with uninfected control mice, both were elevated in three MSS groups which represented *C. albicans*-infected mice and increased with aggravation of sepsis (Figure 3C). These results demonstrate that mTOR is involved in regulating CD4^+^ T cells in *Candida* sepsis.

### Expression of T-Bet in Different Degrees of *Candida* Sepsis Is Regulated by mTOR

**FIGURE 4:**
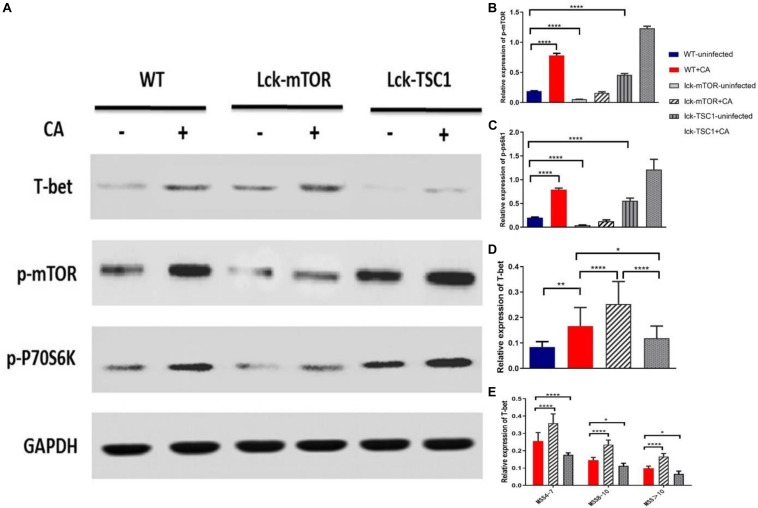
Expression of T-bet in different degrees of *Candida* sepsis is regulated by mTOR. **(A–E)** Protein levels of T-bet, phospho (p)-mTOR and p-P70S6 were quantified by western blotting from sorted CD4^+^ T cells in wild-type (WT), *mTOR* knockout (*Lck-mTOR*) and *TSC1* knockout (*Lck-TSC1*) mice. The amount of each protein level was normalized by GAPDH. Mean ± SD, 10 mice per MSS group (“+CA” groups contained all infected mice of three MSS groups), analyzed by one-way analysis of variance multiple comparisons. *P < 0.05, **P < 0.01, ****P < 0.0001.

To further investigate if the mTOR pathway regulated T-bet expression in *Candida* sepsis, we used T-cell-specific *mTOR* knockout *(Lck-mTOR*) mice and T-cell-specific *TSC1* knockout *(Lck-TSC1*) mice. Firstly, we verified mRNA expression by qPCR as the description in our previous study (Wang et al., 2019). Secondly, we detected the protein level of p-mTOR (phosphorylation at Ser2448) and activity of p70S6 kinase (phosphorylation at Thr389), which were both elevated in *Lck-TSC1* mice and reduced in *Lck-mTOR* mice (Figures 4A–C). These results confirmed the gene knockout effect. Thirdly, we detected T-bet expression in gene knockout mice with *Candida* sepsis (Figures 4A,D). Compared with WT mice, the T-bet expression of mice in the *Lck*-*mTOR* group was significantly increased while that in the *Lck-TSC1* group was reversed, indicating that the expression of T-bet requires mTOR signaling pathway. At last, we compared the difference of T-bet expression between *Lck-mTOR* mice and *Lck-TSC1* mice under the same infection degree (MSS group) and found that in the same MSS group (Figure 4E), *Lck-mTOR* mice had more T-bet expression than WT mice, while *Lck-TSC1* mice had less expression than WT mice. These results indicated that the expression of T-bet is regulated by the mTOR signaling pathway during *Candida* sepsis.

### Variation in CD4^+^ T Cell Count and T-Bet Expression in T-Cell-Specific *mTOR/TSC1* Knockout Mice With Lethal *Candida* Sepsis

**FIGURE 5:**
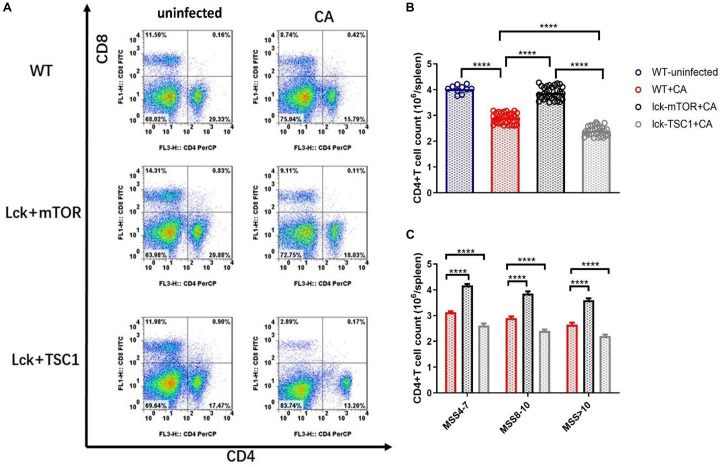
CD4^+^ T cell count during *Candida* sepsis is affected by mTOR pathway. Splenocytes of specific gene knockout mice were obtained at 12 h after *Candida* injection. **(A–C)** Gating strategy for CD4^+^CD8^–^ and CD4^–^CD8^+^ T cells by flow cytometry analysis. Mean ± SD, 10 mice per MSS group (“+CA” groups contained all infected mice of three MSS groups), analyzed by one-way analysis of variance multiple comparisons. ****P < 0.0001.

To further demonstrate that CD4^+^ T cell count is influenced by T-bet expression mediated by mTOR during lethal *Candida* sepsis, we measured the CD4^+^ T cell count of gene knockout mice with *Candida* sepsis (Figures 5A–C). CD4^+^ T cell count and T-bet expression had the same variation tendency in gene knockout mice with lethal *Candida* sepsis. In the same MSS group, *Lck-mTOR* mice had more T-bet expression and CD4^+^ T cell count than WT mice had, while *Lck-TSC1* mice had less than WT mice had. These results demonstrate that the mTOR pathway affects CD4^+^ T cell count by regulating T-bet expression level during *Candida* sepsis.

## Discussion

To our knowledge, this is the first study to demonstrate that expression of CD4-specific transcription factor T-bet regulated by the mTOR signaling pathway influences CD4^+^ T cell count in lethal *Candida* sepsis of different severity.

Invasive candidiasis has been a great challenge in clinical practice, especially in critical care, for the past few decades. Despite our efforts to improve early recognition of candidiasis and to prevent or treat it with antifungal agents, it is still common and lethal in immunosuppressed and critically ill patients. Many studies have proved that compromise of host immunity is one of the main reasons (Spec et al., 2016). As a component of the adaptive immune system, CD4^+^ T cells play a major role in the process of eliminating invasive *Candida* (Seana et al., 2015). *Candida* count has been proved to reflect host immune status (Limper et al., 2017). After *Candida* invades the body, naïve T cells regulated by specific transcription factors begin to differentiate and develop into various effector T helper cells to protect the host against disseminated *Candida* (Romani, 2011). Thus, a decrease in CD4^+^ T cell count may be partly caused by disordered differentiation. In the present study, after mice were infected, the CD4^+^ T cell count decreased significantly and decreased further with aggravation of sepsis, which meant that host immunity was impaired.

T-bet is the main coordinator of gene expression of type 1 immune T cells and a key transcription factor for the differentiation of naïve T cells into Th1 cells. It has been shown that Th1 cells play a central role in eliminating disseminated *Candida*, mainly by releasing interferon-γ, which mediates cellular immunity and induces delayed hypersensitivity (Delsing et al., 2014). Hence, in the process of *Candida* sepsis, T-bet acts on CD4^+^ T cells to regulate the differentiation and growth of its effector cells, thus participating in the clearance of disseminated *Candida*. In this research, we found that T-bet expression increased after infection but decreased with aggravation of sepsis, suggesting that T-bet plays an important role in the regulation of CD4^+^ T cells during lethal *Candida* sepsis. In addition, we found that compared with non-infected mice, the CD4^+^ T cell count of infected mice decreased, but the expression of T-bet significantly increased. We think the reason is infection induces the initiation of immune response, which promotes the differentiation of naive T cells into effector cells and increases the expression of T-bet. However, when severe infection occurs, the severity of infection has a very important and complex influence on the immune functional differentiation in T cells.

For further analysis, we used MSS to confirm the sepsis model and found that mice with different MSS had different survival time. In this study, mice with high MSS had shorter survival time and lower survival rate, which meant that they had more severe sepsis. This finding is consistent with research by Shrum et al. (2014), in which MSS was used in mice with sepsis, to assess the severity of the sepsis model. As a result, we divided the experimental mice into three MSS groups (4–7, 8–10, and >10) to represent different degrees of sepsis and measured CD4^+^ T cell count and T-bet expression. We found that mice in MSS group 4–7 had the highest T-bet expression and CD4^+^ T cell count, while mice in MSS group >10 had the lowest T-bet expression and CD4^+^ T cell count. This result demonstrated that the more severe *Candida* sepsis was, the lower the T-bet expression, indicating less Th1 differentiation during this process. This is consistent with a prospective clinical observational study that demonstrated a significant increase in T-bet expression in the sepsis/severe sepsis/septic shock group compared to the uninfected control group, and a gradual decrease in T-bet expression as the disease progressed (Li et al., 2015). In addition, in our study, the MSS >10 group had the lowest number of CD4^+^ T cells, indicating the most severe immunosuppression and weakest adaptive immune response, which explains why the survival time of this group was the shortest. This finding is consistent with the study of Wu et al. (2013) in which high Th1 differentiation and CD4^+^ T cell count in septic mice had higher survival rates.

However, in the clinical study by Xu et al. (2019), the sepsis group showed decreased T-bet expression compared to the uninfected control group. The reason for this difference may be that, although all patients had sepsis, most of the pathogens were bacteria, which differed from our study, in which the pathogen was a fungus. It is interesting to note that Xu et al. found that patients in the survival group had higher T-bet expression than those that died, suggesting that the higher the severity of sepsis, the lower the expression of T-bet. This is consistent with our results. We believe that comparison of infected and uninfected groups differs between the two studies because different pathogens caused sepsis, which led to emergence of pathogen-specific host immune response at the initial stage of infection. However, as the infection progresses and worsens into sepsis and even septic shock, the host immune response gradually becomes uniform, that is, immune imbalance caused by simultaneous proinflammatory and anti-inflammatory effects. At this stage, the signaling pathways and mechanisms that regulate the host immune response may be dominated by sepsis, so the same results are obtained.

Indeed, the effector differentiation process of T cells during infection is influenced by multiple signaling channels and transcription factors. This is one of the main issues of our study. T-bet expression is regulated by the mTOR signaling pathway (Chornoguz et al., 2017). In our previous studies, we proved that mTOR also affected CD8^+^ T cell differentiation in mice with invasive pulmonary aspergillosis by regulating expression of T-bet and Eomes, and mTOR and its downstream S6k1 levels were positively correlated with T-bet and Eomes expression (Cui et al., 2016). Thus, to further uncover the underlying mechanism, we used western blotting to assess activity of mTOR during this lethal fungal sepsis. mTOR activity significantly increased after infection and further increased with aggravation of sepsis, which indicates that mTOR is involved in regulating CD4^+^ T cells in *Candida* sepsis. After that, we used gene-specific knockout of *mTOR* and *TSC1* mice to strengthen/inhibit the mTOR signaling pathway. During lethal *Candida* sepsis, mice in which the mTOR signaling pathway was inhibited by T-cell-specific mTOR deletion had higher expression of T-bet and CD4^+^ T cell count than WT mice had. Also, mice with T-cell-specific *TSC1* deletion had lower expression of T-bet and CD4^+^ T cell count indicating that the expression of T-bet required mTOR to participate in the regulation, and the regulation affected the CD4^+^ T cell count in *Candida* sepsis. This is not consistent with a recent study that showed that expression of T-bet decreased and Th1 cells could not differentiate after inhibition of mTOR by rapamycin (Chornoguz et al., 2017). In that study, the differentiation of CD4^+^ T cells was achieved in a normal physiological state, while differentiation occurred in a state of lethal *Candida* sepsis in our study. Different pathological conditions may be a major reason for the different results in the two studies. Many studies have shown that proliferation and differentiation of naïve T cells have significant characteristics under different pathological conditions including infection and tumor, or even in specific cytokine environments (Resende et al., 2013; Saleh et al., 2019). In addition, our result showed even in presence of mTOR deficiency there is a severity-dependent decrease of T-bet and CD4^+^ T cell count, this is consistent with the study of Jin et al. (2019). They found that when severe infection occurred, under the regulatory of Notch signaling pathway, CD4^+^ T cell count decreased and continued to decrease with the worsening of sepsis. Thus, we think the change of CD4^+^ T cell count in our study is due to the joint action of different regulatory mechanisms and changes in a single regulatory pathway may not reflect the overall results of immune differentiation, which needs comprehensive consideration. In a future study we aim to clarify the subpopulation in CD4^+^ T cell differentiation, such as CD4 type 17 helper T (Th17) cells (Lin et al., 2009), and the relationship between specific transcription and other possible influencing factors in lethal fungal sepsis, to explore the specific mechanism behind this phenomenon.

In summary, our research provides the first preliminary evidence that T-bet expression mediated by the mTOR pathway influences CD4^+^ T cell count in mice with lethal *Candida* sepsis, which provides new targets and potential therapeutic strategies for improving immune compromise.

## Data Availability Statement

The datasets generated for this study are available on request to the corresponding author.

## Ethics Statement

The animal study was reviewed and approved by the Institutional Animal Care and Use Committee (IACUC) of PUMCH.

## Author Contributions

NC, HW, GB, and WH together participated in the study design and writing the manuscript and experiments.

## Conflict of Interest

The authors declare that the research was conducted in the absence of any commercial or financial relationships that could be construed as a potential conflict of interest.
