# Supplementary material for: T-Bet Expression Mediated by the mTOR Pathway Influences CD4+ T Cell Count in Mice With Lethal Candida Sepsis
Source: Front Microbiol. 2020 May 5;11:835. doi: 10.3389/fmicb.2020.00835 (PMC7214724; doi:10.3389/fmicb.2020.00835)
Supplement: Supplementary file 1 [file Data_Sheet_1.PDF]

Supplementary Table 1. Parameters of MSS

Variable	Score and description
Appearance	0- Coat is smooth
	1- Patches of hair piloerected
	2- Majority of back is piloerected
	3- Piloerection may or may not be present, mouse appears "puffy"
	4- Piloerection may or may not be present, mouse appears emaciated
Level of consciousness	0-Mouse is active
	1- Mouse is active but avoids standing upright
	2- Mouse activity is noticeably slowed. The mouse is still ambulant.
	3- Activity is impaired. Mouse only moves when provoked, movements have a tremor
	4- Activity severely impaired. Mouse remains stationary when provoked, with possible tremor
Activity	0- Normal amount of activity. Mouse is any of: eating, drinking, climbing, running, fighting
	1- Slightly suppressed activity. Mouse is moving around bottom of cage
	2- Suppressed activity. Mouse is stationary with occasional investigative movements
	3- No activity. Mouse is stationary
	4- No activity. Mouse experiencing tremors, particularly in the hind legs
Response to stimulus	0- Mouse responds immediately to auditory stimulus or touch
	1- Slow or no response to auditory stimulus; strong response to touch (moves to escape)
	2- No response to auditory stimulus; moderate response to touch (moves a few steps)
	3- No response to auditory stimulus; mild response to touch (no locomotion)
	4- No response to auditory stimulus. Little or no response to touch. Cannot right itself if pushed over
Eyes	0- Open
	1- Eyes not fully open, possibly with secretions
	2- Eyes at least half closed, possibly with secretions
	3- Eyes half closed or more, possibly with secretions
	4- Eyes closed or milky
Respiration rate	0- Normal, rapid mouse respiration
	1- Slightly decreased respiration (rate not quantifiable by eye)
	2- Moderately reduced respiration (rate at the upper range of quantifying by eye)
	3- Severely reduced respiration (rate easily countable by eye, 0.5 s between breaths)
	4- Extremely reduced respiration (>1 s between breaths)
Respiration quality	0- Normal
	1- Brief periods of laboured breathing
	2- Laboured, no gasping
	3- Laboured with intermittent gasps
	4- Gasping

(Shrum B, et al. BMC research notes 2014; 7(1):233.)

Supplementary Figure 1. Example for the gating strategy in FCM.

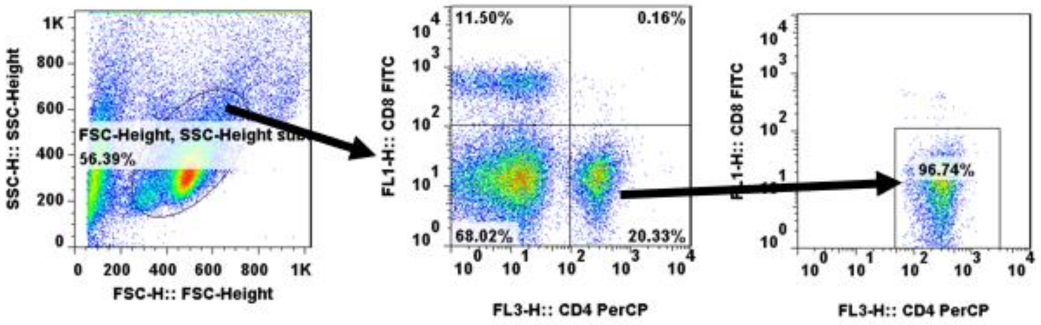

Flow cytometry gating strategy for CD4+T cells subsets in the whole PBMCs of spleen. Based on the PBMCs of spleen, we detected the ratio of CD4+ T cells. The PBMCs were initially gated by forward and side scatter properties (left panel). CD4+ T-cells and CD8+ T-cells (middle panel) were then gated. Then we sorted CD4+ T cells and the purity of CD4+ T cells (right panel) was also detected. The sorted CD4+ T cells were then used for western blotting. The machine we used is BD FACS AriaIII.

Supplementary Figure 2. Shapiro-Wilk Test for CD4+T cell count and T-bet expression in each group.

Tests of Normality

		Kolmogorov-Smirnov[a]			Shapiro-Wilk		
	Group	Statistic	df	Sig.	Statistic	df	Sig.
WTtbet	1	.104	10	.200*	.966	10	.847
WTCAtbet	1	.212	10	.200*	.884	10	.144
mTORCAtbet	1	.169	10	.200*	.979	10	.960
TSC1CAtbet	1	.138	10	.200*	.920	10	.354
WTCACD4	1	.178	10	.200*	.926	10	.408
mTORCACD4	1	.123	10	.200*	.966	10	.856
TSC1CACD4	1	.143	10	.200*	.933	10	.481

*. This is a lower bound of the true significance.

a. Lilliefors Significance Correction

SPSS was used for performing the Shapiro-Wilk test for $CD4^{+}$T cell count and T-bet expression in each group, and it can be seen from the figure that all the Sig. of each group was >0.05, which verified the data was normality distribution.

Supplementary Figure 3. Summarization of the flow cytometry scatter plots.

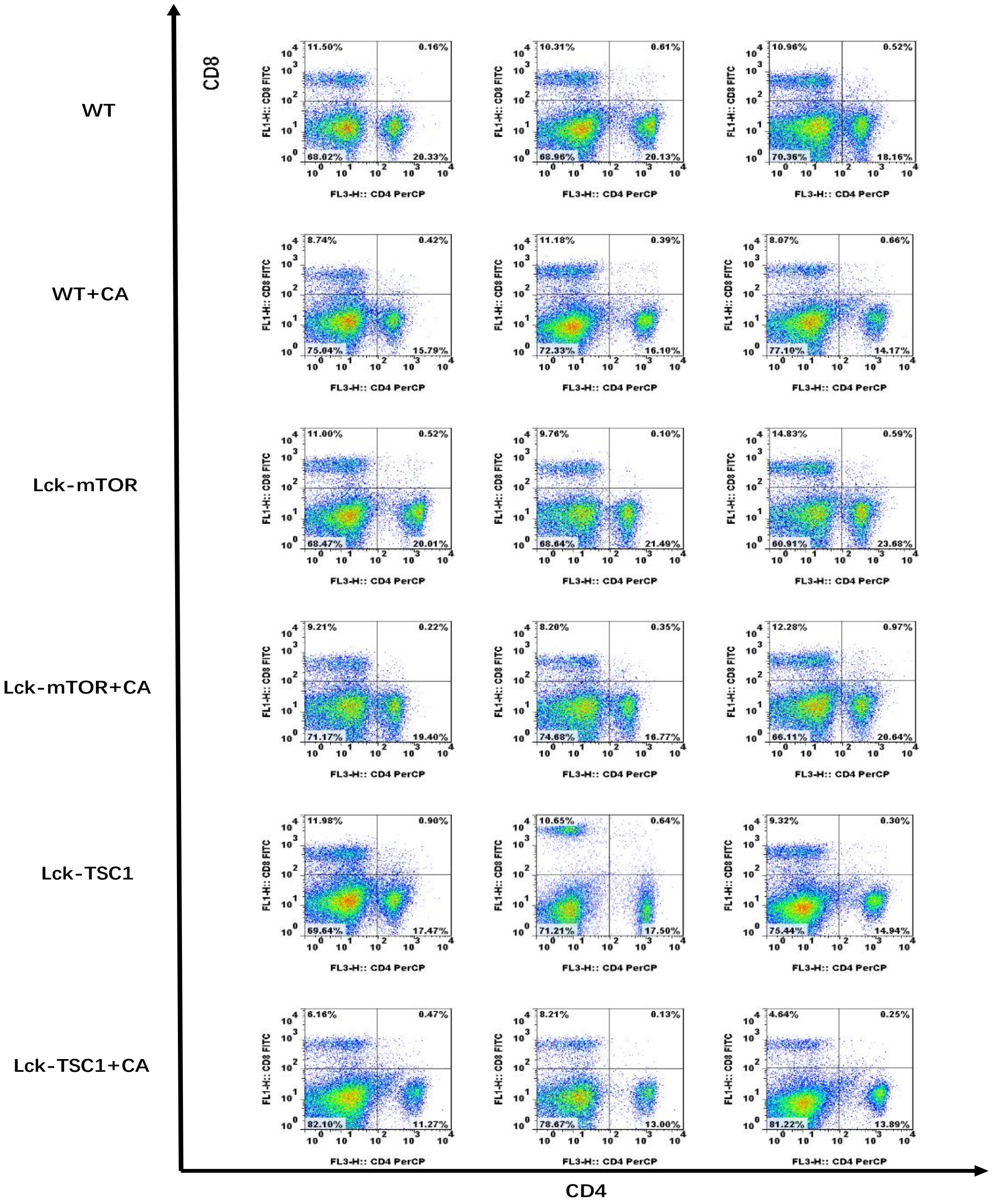

Supplementary Figure 4. Graph for percentage of $CD4^{+}CD8^{-}$T cells and $CD4^{-}CD8^{+}$T cells of the whole lymphocyte

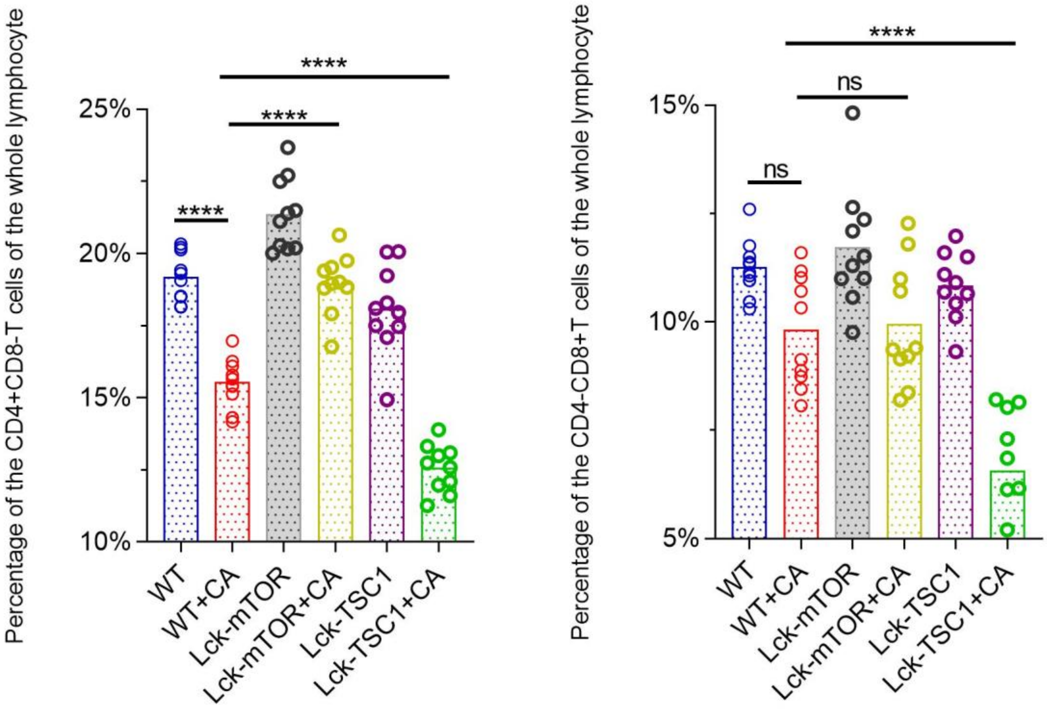

GraphPad Prism version 8 was used for performing this graph. One-way analysis of variance (ANOVA) followed by Bonferroni's test were used to determine the statistical significance of differences. $P < 0.05$ was considered statistically significant. ****$P < 0.0001$. ns= no significance.
